# Clinical Progress of Fruquintinib in Colorectal Cancer: An Overview

**DOI:** 10.3390/ph18020280

**Published:** 2025-02-19

**Authors:** Yejie Xie, Shu Tang, Ziheng Qin, Chaogang Yang

**Affiliations:** 1 The Second Clinical School of Wuhan University, Wuhan 430071, China; 2021302131065@whu.edu.cn (Y.X.);; 2 Department of Anesthesia and Surgery, Zhongnan Hospital of Wuhan University, Wuhan 430071, China; 3 Department of Gastrointestinal Surgery, Zhongnan Hospital of Wuhan University, Hubei Key Laboratory of Tumour Biological Behaviours, Hubei Cancer Clinical Study Centre, The Clinical Medical Research Centre of Peritoneal Cancer of Wuhan, Wuhan 430071, China

**Keywords:** fruquintinib, colorectal cancer, VEGFR, clinical trial, adverse event

## Abstract

Colorectal cancer (CRC) is one of the most common malignancies worldwide, with high morbidity and mortality rates. Conventional treatments, including surgery, radiotherapy, and chemotherapy, have limited effects on advanced and metastatic CRC (mCRC). Fruquintinib, a novel and highly selective vascular endothelial growth factor receptor (VEGFR) inhibitor, has shown significant efficacy and tolerance in treating mCRC. The FRESCO and FRESCO-2 trials demonstrated that fruquintinib significantly prolongs progression-free survival and the overall survival of refractory mCRC patients, establishing it as the standard third-line treatment strategy for mCRC. In addition, the combination of fruquintinib with other anticancer drugs and immune checkpoint inhibitors demonstrated potential for enhanced efficacy, which warrants further exploration. In this review, we aimed to systematically summarize the current knowledge about the pharmacological mechanisms, pharmacokinetic characteristics, adverse events, and corresponding treatment options of fruquintinib and provide an update on the clinical trials related to fruquintinib in CRC by conducting a comprehensive literature search of PubMed and consulting the relevant clinical trials via ClinicalTrials.gov and the ChiCTR website, aiming to offer new insights into the role of fruquintinib in the comprehensive treatment of CRC.

## 1. Introduction

Colorectal cancer (CRC) is one of the most common malignant tumors worldwide and a leading cause of cancer-related deaths. According to the latest global cancer statistics, CRC ranks in third place in terms of incidence but second in terms of mortality, with more than 1.9 million new cases and 904,000 deaths occurring in 2022 [1]. The high incidence and mortality rates of CRC have attracted worldwide attention. In China, the incidence and mortality rates of CRC have shown an increasing trend. In 2022, there will be approximately 517,100 new cases of CRC and 240,000 deaths [2,3]. Despite improvements in the five-year relative survival rate, challenges remain. According to a systematic review and meta-analysis, the one-, three-, and five-year survival rates of CRC patients were 79%, 72%, and 62%, respectively [3]. These data reflect some progress in the early diagnosis and treatment of CRC in China but also underscore the need for further advancements.

At present, clinical treatment options for CRC are divided into first-, second-, and third-line treatments based on disease progression. For patients with early-stage CRC, surgical resection is the preferred treatment, and supplementation with radiotherapy and chemotherapy can effectively reduce the risk of recurrence. In contrast, for patients with advanced CRC, especially those who have already developed metastases, treatment becomes significantly more difficult. The standard first-line regimen usually consists of FOLFOX (oxaliplatin in combination with fluorouracil and calcium folinic acid) or FOLFIRI (irinotecan in combination with fluorouracil and calcium folinic acid) alongside targeted therapeutic agents like bevacizumab or cetuximab [4,5]. However, metastatic CRC (mCRC) is usually refractory to chemotherapy, resulting in limited therapeutic efficacy and lower survival rates [6,7]. Second-line therapy is administered after first-line failure or disease progression, often involving changes in chemotherapy regimens, such as switching from FOLFOX to FOLFIRI or adding other targeted agents like regorafenib (a multi-targeted tyrosine kinase inhibitor) or abciximab [8].

To enhance the therapeutic effect of mCRC, targeted therapy and immunotherapy as third-line treatment options have gradually become research focal points. Targeted therapy inhibits the growth and spread of cancer cells by interfering with specific molecular pathways. Common targeted drugs for mCRC include anti-epidermal growth factor receptor (EGFR) monoclonal antibodies like cetuximab and anti-angiogenic drugs like bevacizumab. Although these drugs can improve patients’ progression-free survival (PFS) and overall survival (OS) to some extent, they face challenges with drug resistance and adverse events (AEs) [6]. Immunotherapy is another cutting-edge treatment that attacks cancer cells by activating the patient’s immune system. Programmed death protein 1 (PD-1) and programmed death ligand 1 (PD-L1) inhibitors have demonstrated significant efficacy in a wide range of cancers, including CRC [9]. However, the effectiveness of immunotherapy in CRC has been limited, primarily benefiting patients with high microsatellite instability (MSI-H) or defective mismatch repair (dMMR). For these patients, immune checkpoint inhibitors like nivolumab and pembrolizumab are effective, but broader application in CRC requires more research [10]. Besides targeted therapy and immunotherapy, novel chemotherapeutic agents and combination therapies are also being explored. Recently, oral fluorouracil analogs such as S-1 and capecitabine have been clinically applied, showing good efficacy and fewer AEs. Combining traditional chemotherapeutic drugs with targeted or immunotherapeutic drugs is an effective strategy [10].

Based on these therapeutic strategies [4,5,6,7,8,9,10], a novel targeted drug, fruquintinib, has emerged. Fruquintinib (also known as HMPL-013), first discovered by Hutchison Whampoa Pharmaceuticals Limited in 2005, is a selective vascular endothelial growth factor receptor (VEGFR) inhibitor [11]. It has shown significant anti-tumor activity and good tolerance in several clinical trials, including the phase III FRESCO and FRESCO-2 trials, which demonstrated that fruquintinib could significantly improve the PFS and OS for refractory mCRC patients [11,12]. Based on the above pivotal data, the Chinese National Medical Products Administration (NMPA) and the US Food and Drug Administration (FDA) have granted approval for fruquintinib for the treatment of mCRC patients who have failed at least two prior systemic anti-tumor therapies. At present, multiple clinical trials on fruquintinib in first- or second-line treatment for mCRC and neoadjuvant therapy for locally advanced CRC are also in full swing, expecting to provide more high-level evidence for the application of fruquintinib in the comprehensive treatment of CRC. Furthermore, the safety of fruquintinib has also been reported to be controllable while achieving good clinical efficacy [13].

In the current review, we aimed to systematically summarize the current knowledge about the pharmacological mechanisms, pharmacokinetic characteristics, AEs, and corresponding treatment options, as well as the latest clinical trials related to CRC by conducting a comprehensive literature search in PubMed and consulting the relevant clinical trials via ClinicalTrials.gov and the ChiCTR website, aiming to offer new insights into the role of fruquintinib in the comprehensive treatment of CRC.

## 2. Angiogenesis, Fruquintinib, and CRC

Angiogenesis plays a critical role in normal physiological conditions, such as wound healing and embryonic development. However, in CRC, excessive angiogenesis is often closely associated with the incidence and extent of metastasis. The neovascularization hypothesis, first proposed by Judah Folkman, suggests that abnormal angiogenesis provides nutrients and oxygen to the tumor, as well as metastatic pathways [14]. Typically, angiogenesis is regulated by the vascular endothelial growth factor (VEGF) and its receptor (VEGFR) [15], which promotes angiogenesis by stimulating endothelial cell proliferation and migration. Previous studies have shown that high levels of VEGF expression in CRC generally indicate a worse patient prognosis [16].

Among VEGFR family members (VEGFR-1, 2, and 3), VEGFR-1 (also known as Flt-1) plays a relatively weak role in signaling due to its low tyrosine kinase activity [17]. In contrast, VEGFR-2 (also known as KDR/Flk-1) primarily mediates VEGF signaling and has higher tyrosine kinase activity, which drives various functions of vascular endothelial cells [18]. When VEGF binds to VEGFR-2, it activates a variety of downstream signaling pathways, including RAS/MAPK, PI3K/AKT, and PLCγ, thereby promoting angiogenesis and tumor progression [19]. VEGFR-3 (Flt-4) is primarily involved in lymphangiogenesis [20], with VEGF-C and VEGF-D as main ligands, which plays a crucial role in regulating lymphatic endothelial cell functions and has become a significant focus of research in cancer studies [15].

Currently, there are two main approaches targeting the VEGF/VEGFR axis as anticancer therapy: monoclonal antibodies that neutralize or inhibit VEGF and small-molecule inhibitors that block VEGFR kinase activity [21]. Bevacizumab, the first anti-VEGF monoclonal antibody approved for anticancer therapy, works by binding to VEGF and blocking its interaction, thereby inhibiting tumor angiogenesis [22]. Nowadays, bevacizumab, in combination with 5-fluorouracil-based chemotherapy, has been approved as a first- or second-line treatment option for mCRC [23]. However, long-term bevacizumab treatment can lead to drug resistance, hypertension, proteinuria, and thrombosis, along with high costs [7]. Additionally, early small-molecule VEGFR inhibitors like sunitinib [24], sorafenib [25], regorafenib [26], and pazopanib [27] inhibit multiple kinases with poor selectivity, resulting in limited drug exposure at the maximum tolerated dose (MTD) and shorter inhibition duration for VEGFR (Table 1).

**Figure 1:**
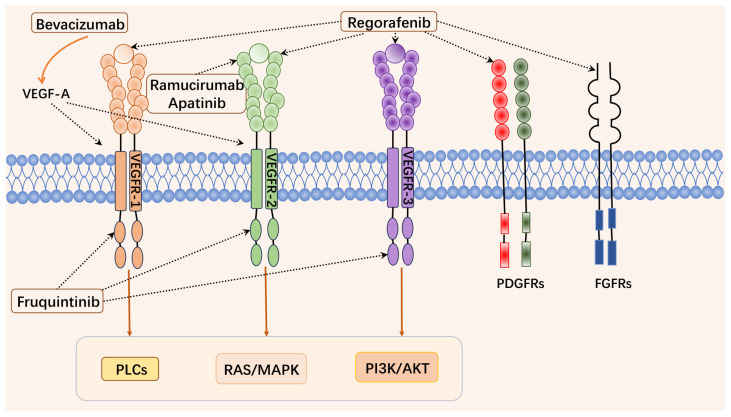
The targets and related signaling pathways of anti-angiogenic drugs in CRC.

Fruquintinib (6-(6,7-dimethoxyquinazolin-4-yloxy)-N, 2-dimethylbenzofuran-3-carboxamide) is a new-generation small-molecule tyrosine kinase inhibitor (TKI) [32], which is a class of targeted therapy drugs designed to inhibit the activity of tyrosine kinases—enzymes that play a critical role in cellular signaling pathways. These enzymes are often implicated in the growth, proliferation, and survival of cancer cells. By blocking the action of tyrosine kinases, TKIs can disrupt signaling pathways that drive tumor progression, making them a key therapeutic option in the treatment of various cancers, particularly those driven by specific genetic mutations or overactive kinase activity [32]. As a novel TKI with highly selective and potent inhibition of VEGFR-1, -2, and -3, fruquintinib effectively limits tumor growth by inhibiting angiogenesis and tumor blood vessel maturation [32]. Its mechanism reduces off-target toxicity and provides a high level of drug exposure at the MTD for sustained VEGFR inhibition, which can be used alone or with other therapeutic agents for a comprehensive anti-tumor effect (Figure 1).

## 3. Pharmacodynamic and Pharmacokinetic Characteristics of Fruquintinib

In the experiments assessing 253 kinases for selectivity, fruquintinib was found to inhibit VEGFR-1, -2, and -3 with half-inhibitory concentrations (IC50 values) of 33, 35, and 0.5 nmol/L, respectively. By contrast, it was less active against kinases like RET, FGFR-1, and c-kit (with IC50 values of 128–458 nmol/L) and showed little or no inhibition of the other kinases tested (the IC50 values all exceeded 1000 nmol/L) [33].

Fruquintinib competitively binds to the kinase domain of VEGFR, effectively preventing VEGF from binding to and activating its receptor. This interaction blocks the conformational changes and dimerization of VEGFR, thereby inhibiting phosphorylation of the receptor’s kinase structural domains within the cell. As a result, VEGF-dependent downstream signaling pathways (e.g., PI3K/AKT, PKC, RAF/RAS, and ERK pathways) for cell proliferation, migration, and neo-angiogenesis are disrupted. The experimental data have shown that fruquintinib suppressed the tube length of primary cultured human umbilical vein endothelial cells by 74% and 94% at concentrations of 0.03 and 0.3 mmol/L, respectively; however, this did not have a significant inhibitory effect in a separate cell survival experiment, suggesting that fruquintinib’s inhibitory effect is primarily achieved through the VEGF/VEGFR axis rather than direct cytotoxicity. In addition, the follow-up experiments demonstrated a significant dose-dependent effect of fruquintinib [33].

Angiogenesis is an important component of the tumor microenvironment (TME). Studies have shown that fruquintinib can normalize tumor vasculature and inhibit the formation of new tumor blood vessels by targeting VEGFR. This, in turn, improves the hypoxic condition of tumor cells and optimizes the TME [34]. Such effects suggest that fruquintinib might have immune-enhancing potential and can be used in combination therapy. In a study investigating the protective effects of fruquintinib on choroidal neovascularization in mice, it was found that fruquintinib downregulated the expression levels of relevant markers (IL-6, TNF-α, RANTES, and VEGF/VEGFR-2) in M1-type macrophages under hypoxic conditions. This inhibition of VEGF/VEGFR-2-induced polarization of M1-type macrophages under hypoxic highlights its potential therapeutic benefits [35].

In both in vitro experiments and tumor xenograft models, fruquintinib has shown the ability to inhibit a wide range of human tumors, including gastric, colon, and lung cancers. In patient-derived xenograft (PDX) models of gastric and colon cancers, enhanced anti-tumor activity was observed when fruquintinib was combined with doxorubicin and oxaliplatin, resulting in a reduction in tumor growth inhibition by approximately 30% [33]. In addition, low-dose fruquintinib in combination with an anti-PD-1 antibody appeared to enhance the anti-tumor immune response, reduce angiogenesis, increase the infiltration of CD8+ T cells, decrease the proportion of immunosuppressive cells such as macrophages and myeloid-derived suppressor cells (MDSCs), and improve the tumor immune microenvironment [34]. In a lung cancer model, the anti-tumor effect of fruquintinib was enhanced and could reduce the tumor volume by more than 85% when used in combination with other targeted agents, such as c-MET inhibitors and TKIs, suggesting a combination therapy of its great potential [36].

Fruquintinib exhibits an excellent linear pharmacokinetic profile in Chinese patients with solid tumors. After a single oral dose of 1–6 mg, it is rapidly absorbed, and systemic exposure increases proportionally with the dose, with small inter-individual variations [13]. At all dose levels, Cmax is reached 2.0–4.0 h post-administration. Fruquintinib is a small molecule with slow elimination, high plasma protein binding (~95%), and poor tissue distribution properties [36]. These properties allow it to maintain high exposure levels in the plasma and have a long elimination half-life (T1/2~42 h). In a study of healthy Chinese males receiving a dose of radiolabeled fruquintinib, it was found that the unmetabolized drug dominated the plasma-associated fractions, accounting for 93.33%, 94.77%, 88.54%, and 48.41% of the total radioactivity at 0.5, 2, 8, and 96 h, respectively [37]. Additionally, fruquintinib is metabolized hepatic mainly via CYP3A, with lesser contributions from CYP2C8, CYP2C9, and CYP2C19. After metabolism, most metabolites are excreted via urine, whereas only small amounts of the unmetabolized parent drug are excreted via urine (0.5%) or feces (5%) [10]. Taken together, fruquintinib exhibits the ability to inhibit a wide range of human tumors in in vitro experiments and tumor xenograft models, making it a promising candidate for combination therapy. Its favorable pharmacokinetic profile, including low absorption variability, high plasma protein binding rate, and long elimination half-life, suggests broad applicability in anti-tumor therapy.

## 4. Clinical Trials of Fruquintinib for CRC

The anti-tumor activity of fruquintinib has been extensively demonstrated in several clinical trials, particularly in the treatment of patients with chemotherapy-resistant mCRC. Among these, several key clinical trials are presented in Table 2.

### 4.1. Phase I

A phase I clinical trial conducted by Cao et al. was an open, dose-escalation, dose-expansion study designed to determine the safety and MTD of fruquintinib and to recommend a phase II dose (RP2D) for future clinical trials. In addition, this study evaluated the pharmacokinetic properties of fruquintinib and explored its anti-tumor activity in patients with advanced solid tumors. The study was conducted in China and involved 40 patients with various tumor types, all of whom had undergone rigorous systemic anticancer pretreatment. Patients were divided into five continuous treatment groups (1–6 mg) and two 3-week on/1-week off groups (5 and 6 mg). Dose-limiting toxicity, primarily in the form of skin reactions of the hands and feet, was observed in the 6 mg continuous treatment group, while symptomatic thrombocytopenia and hand-foot syndrome (HFS) reactions were noted in the 5 mg continuous treatment group. Based on these results, 4 mg was determined to be the optimal dose for continuous treatment. In the 3-week on/1-week off regimen, a grade 3 fatigue reaction occurred in the 6 mg group. Considering the long-term use of fruquintinib and patient tolerability, a regimen of 5 mg per day 3-week on/1-week off regimen is recommended for further efficacy and safety trial studies [13]. Although this dosage has been proven to be relatively safe and effective, a key focus of future research will be determining how to further reduce adverse effects without compromising efficacy.

Additionally, an open, single-arm, phase Ib trial was conducted between December 2012 and January 2014 at two hospitals in China (NCT01975077). The study involved patients with mCRC who had disease progression despite three months of at least two prior treatment regimens, including fluoropyrimidine, oxaliplatin, or irinotecan. The study employed a regimen of 5 mg per day for a 3-week on/1-week off cycle, with PFS as the primary endpoint. A total of 42 patients aged between 33 and 70 years were enrolled, with 31 (73.8%) patients completing at least three treatment cycles within 12 weeks and 28 (66.7%) patients completing at least four treatment cycles within 16 weeks. The study results showed a median PFS of 5.80 months (95%CI: 4.01–7.60) and a median OS of 8.88 months (95%CI: 7.53–15.53). The disease control rate (DCR) and objective remission rate (ORR) were 76.2% and 9.5%, respectively. All 42 patients experienced treatment-related AEs, with the most common grade 3 or higher AEs being hypertension (21.4%), hand-foot-skin reactions (9.5%), and diarrhea (9.5%). Overall, five patients permanently discontinued the drug due to related adverse reactions, including skin toxicity, chest pain, hemoptysis, pancreatitis, and proteinuria. One case of death from hemoptysis due to pulmonary metastases has been reported [38].

### 4.2. Phase II

A randomized, double-blind, placebo-controlled phase II trial (NCT02196688) was conducted from April to August 2014 at eight hospitals in China to assess the efficacy and safety of fruquintinib in combination with best supportive care (BSC) in patients with mCRC who had previously received multiple treatments. A total of 71 patients were randomized 2:1 to fruquintinib plus BSC or the placebo plus BSC. The baseline characteristics of the patients in both groups were balanced. The fruquintinib group had a treatment period of 3.2 months, compared to 0.8 months for the placebo group. The primary endpoint was PFS. Patients in the fruquintinib group had a significantly prolonged PFS compared to those in the placebo group (median PFS, 4.73 months; stratified HR, 0.30; 95%CI: 0.15–0.59; *p* < 0.001). Although there were no significant differences in ORR and OS, the DCR was higher in the fruquintinib group. In addition, the incidence of adverse reactions, hypertension (29.8%), and hypertensive cerebral hemorrhage (14.9%) were significantly higher in the fruquintinib group (93.6%) than in the placebo group (58.3%). Serious adverse reactions occurred in 12 (25.5%) patients in the fruquintinib group compared to 5 (20.8%) in the placebo group. Dose adjustments were needed in 61.7% of fruquintinib patients and 16.7% of the placebo group [38].

An open, single-arm, phase II trial (NCT05025631) was also conducted in patients with mCRC aged 65 years or older who had disease progression despite having received at least two standard chemotherapies. Most patients (93.1%) had been previously treated with bevacizumab or cetuximab. The trial was designed as a 28-day treatment cycle starting with 21 consecutive days of fruquintinib at 3 mg/day in the first week, 4 mg/day in the second week, and 5 mg/day in the third week, with adjustments from the second cycle based on the maximally tolerated dose from the first cycle. The primary endpoint was PFS, and the secondary endpoints were OS, safety, and ORR. A total of 29 patients were enrolled in the study, with a median PFS of 3.8 months (95%CI: 2.7–4.9) and a median OS of 7.6 months (95%CI: 6.5–8.7). Common adverse reactions included malaise (79.3%), loss of appetite (58.6%), and HFS (44.8%). Grade 3 or higher AEs occurred in 55.2% of the patients, and four patients discontinued treatment due to AEs. This study is the first to evaluate the efficacy and safety of an initial dose escalation strategy for fruquintinib in elderly patients with multiple refractory CRC [39].

Both studies demonstrated the potential efficacy and safety of fruquintinib in mCRC patients. A randomized, double-blind, placebo-controlled phase II trial identified significant benefits of fruquintinib in prolonging PFS, while the single-arm phase II trial evaluated an initial dose escalation strategy of fruquintinib in elderly patients, providing important safety and efficacy data. Overall, fruquintinib showed promising anti-tumor activity, but its use requires careful consideration of AEs and patient tolerability.

### 4.3. Phase III

The FRESCO study, a phase III randomized, double-blind, placebo-controlled, multicenter trial, evaluated the efficacy and safety of fruquintinib in mCRC patients. The study was conducted at 28 hospitals in China and was designed to provide a new treatment option for patients with mCRC who had poor outcomes after standard treatment. A total of 416 patients were randomized (2:1) to receive fruquintinib plus BSC or the placebo plus BSC from December 2014 to June 2017. The disease characteristics of the two groups were better balanced. The primary endpoint was OS. The final results showed that patients in the fruquintinib group achieved a median OS of 9.30 months, significantly longer by 2.73 months, with a 35% reduction in death risk compared to the placebo group (*p* < 0.001). However, 15.5% of fruquintinib patients experienced serious AEs, with two cases of G5-stage AEs in the placebo group. Subgroup analyses showed a significant benefit in the fruquintinib group with a favorable safety profile and manageable AEs, regardless of whether patients had received prior anti-VEGF or anti-EGFR therapy [28].

Expanding on the FRESCO study, the FRESCO-2 study is a global, randomized, double-blind, placebo-controlled, multicenter phase III trial designed to evaluate the efficacy and safety of fruquintinib in a broader patient population, which included 691 patients from the US, Europe, Australia, and Japan from September 2020 to December 2021. The enrolled patients were randomized in a 2:1 ratio to receive either a regimen of oral fruquintinib 5 mg/dose for 3 weeks on/1 week off or a placebo. In the study population, 96% of the patients (n = 666) had received prior anti-VEGF therapy, and 39% (n = 268) had received prior anti-EGFR therapy. All patients were treated with trifluridine and tipiracil (n = 361, 52%), regorafenib (n = 58, 8%), or both (n = 272, 39%). The primary endpoints were OS and PFS. Compared to the placebo, fruquintinib reduced the risk of death by 34% and significantly prolonged OS (7.4 vs. 4.8 months; HR = 0.66; 95%CI: 0.55–0.80; *p* < 0.001) and PFS (3.7 vs. 1.8 months; HR = 0.32; 95%CI: 0.27–0.39; *p* < 0.001). Grade 3 or higher serious AEs occurred in 63% of the patients treated with fruquintinib compared with 50% of the placebo group who experienced grade 2 or higher serious AEs. The OS plots showed that the proportion of patients still alive 9 months after taking the drug was 41% (95%CI: 36–46) compared with 28% (95%CI: 22–34) in the placebo group [40]. In subsequent subgroup analyses, fruquintinib was found to significantly extend survival compared to the placebo, with consistent efficacy observed across various factors, including age, gender, ECOG performance status, race, RAS status, and prior treatments. This indicates the broad applicability of fruquintinib across diverse patient populations. Notably, the subgroup analysis based on liver metastasis status revealed that fruquintinib demonstrated significant efficacy in both patients with and without liver metastases. This finding is particularly important, as liver metastasis typically correlates with a poorer prognosis. However, fruquintinib’s remarkable performance in this high-risk group underscores its potential to improve outcomes.

The results of both the FRESCO and FRESCO-2 studies demonstrated significant clinical benefit and an acceptable safety profile for fruquintinib in patients with mCRC. The FRESCO study demonstrated the efficacy of fruquintinib in Chinese patients, while the FRESCO-2 study further supports fruquintinib as a new global oral CRC treatment agent of choice, providing an important therapeutic tool for patients with refractory mCRC.

### 4.4. Real-World Study

Two real-world studies based on Chinese patients compared the efficacy and safety of fruquintinib and regorafenib in the treatment of mCRC, intending to provide more data to support the use of these two drugs in practical clinical applications. The first study showed a median PFS of 5.4 months (95%CI: 4.84–5.96) in the fruquintinib arm, with 29.3% of patients achieving stable disease (SD) status. This result is generally consistent with previous clinical trials. Treatment-related grade 3 AEs included hand and foot skin reactions, fatigue, and stomatitis (6% for each), and no grade 4 AEs were observed [41]. The second study involved 105 patients who had multiple CRCs and compared the efficacy and safety of fruquintinib and regorafenib. The results showed that fruquintinib was similar to regorafenib in terms of OS and PFS; however, the DCR (65.3% vs. 54.2%) and ORR (6.1% vs. 2.0%) were higher. Combination immunotherapy showed synergistic effects, with both combination anti-PD-1 treatment groups having a longer mean survival than the monotherapy group. In addition, it was found that OS was significantly longer in patients treated with regorafenib, followed by fruquintinib than in those treated in reverse order (15.0 vs. 8.3 months). In terms of AEs, the incidence of HFS was higher in patients treated with regorafenib than fruquintinib, while patients in the fruquintinib group were more likely to develop grade 3 hypertension [42].

### 4.5. Ongoing Clinical Trials

To date, multiple studies continue to explore the treatment and use of fruquintinib in CRC (Table 3). A multicenter, single-arm, open-label phase II study (NCT06234007) was designed to evaluate the efficacy and safety of SCRT sequential fruquintinib in combination with adebrelimab and CAPOX (oxaliplatin + capecitabine) in the treatment of patients with high-risk locally advanced CRC. In mCRC, several phase III studies are evaluating the effect of fruquintinib monotherapy (NCT04296019, NCT04580785). Many phase II studies are exploring new regimens of combination immunotherapy with different drugs, such as capecitabine in combination with fruquintinib for first-line treatment (NCT06115733) and the efficacy and safety of fruquintinib plus FOLFIRI for second-line treatment (NCT05842525), and other drugs include bevacizumab, sintilimab, camrelizumab, and others. Additionally, studies are assessing the efficacy of fruquintinib in various genetic mutation contexts. For instance, a phase II trial is investigating the efficacy and safety of fruquintinib combined with FOLFIRI for second-line treatment in patients with RAS-mutant mCRC (NCT04593369). Another study evaluates the efficacy and safety of Disitamab Vedotin combined with fruquintinib in patients with HER-2 expression or mutation who have failed at least two standard treatments for mCRC (NCT04744831). In non-CRC cancers, fruquintinib has also shown potential. For example, in advanced gastric cancer (GC), the combination of fruquintinib with paclitaxel for second-line treatment significantly improved PFS in Chinese patients with advanced gastric or gastro-oesophageal junction adenocarcinoma (NCT03223376, the FRUTIGA trial) [43]. Additionally, a trial is evaluating the combination of fruquintinib with SOX as a neoadjuvant treatment for patients with locally advanced GC (NCT05122091). In the first-line treatment of advanced GC, a phase Ib/II trial is assessing the efficacy of combining fruquintinib, teraplizumab, and SOX (NCT05024812).

## 5. Common Adverse Events and Treatment Options

In two multicenter phase III clinical trials, FRESCO (Chinese population) and FRESCO-2 (global population) not only demonstrated significant anti-tumor activity but also provided a systematic evaluation of its associated AEs. Although fruquintinib shows good tolerability, certain AEs are common and need to be managed appropriately.

### 5.1. Hypertension

Hypertension is one of the common AEs of fruquintinib, likely due to the reduction in vascular endothelial production after the inhibition of the VEGFR signaling pathway, leading to vasoconstriction and increased peripheral resistance. In the FRESCO study, the incidence of all grades of hypertension in the fruquintinib group was 55.4%, with a 21.2% incidence of grade ≥ 3 hypertension [44]. Hypertension tends to appear about 10 days after administration of the drug and is usually well controlled with conventional antihypertensive therapy. Strategies for managing hypertension include controlling blood pressure to the desired level before treatment and regular monitoring during treatment. Mild hypertension (grade 1) generally does not require any adjustment in treatment, but moderate to severe hypertension (grades 2 and 3) may require appropriate pharmacological interventions and suspension of therapy until blood pressure returns to safe levels. For life-threatening hypertension (grade 4), immediate and permanent medication suspension and emergency treatment are required. Patients are also advised to make lifestyle modifications, such as reducing salt intake, avoiding smoking and alcohol consumption, and increasing moderate exercise to help control blood pressure.

### 5.2. Hand-Foot Syndrome

HFS is another common AE during fruquintinib therapy, with an incidence of 49.3% for all grades, including 10.8% for grades ≥ 3. This AE is primarily due to an inadequate repair of skin friction damage, capillary damage, and inflammation resulting from VEGFR and PDGFR inhibition. For mild reactions (grade 1), supportive measures such as using moisturizers, topical steroid ointments (e.g., hydrocortisone), and avoiding friction on the hands and feet, pressure, and exposure to hot objects can be effective. For moderate (grade 2) and severe (grade 3) reactions, temporary suspension of the medication and dosage adjustments are recommended. Permanent discontinuation of the medication may be necessary in the case of repeated severe reactions [45].

### 5.3. Proteinuria

Proteinuria is another common AE associated with fruquintinib, occurring in 42.1% of patients, with grade ≥ 3 proteinuria occurring in 3.2% of cases. Proteinuria mostly appeared about 20 days after drug administration, and grade 3 proteinuria could be restored to a grade 1 or pre-dose level after a dose adjustment and active symptomatic treatment. This AE is thought to be related to increased glomerular basement membrane permeability caused by VEGFR inhibition. Relevant management measures include close monitoring of urinary protein levels and, if necessary, suspending the drug until urinary protein is reduced to a safe level. For severe or persistent proteinuria, particularly in patients with nephrotic syndrome, permanent discontinuation of the drug and further renal evaluation and treatment may be required.

### 5.4. Other AEs

In addition, fruquintinib may also cause some other AEs, such as diarrhea, fatigue, loss of appetite, nausea and vomiting, ECG abnormalities, and so on. Although the incidence of these AEs is relatively low, they still need to be taken seriously by clinicians. Attention should be paid to their occurrence, especially in elderly patients. In clinical practice, physicians should be fully aware of the characteristics of fruquintinib’s AEs and develop individualized treatment plans to manage them effectively.

## 6. Conclusions and Perspectives

The application of fruquintinib brings new hope for CRC treatment. As a highly selective VEGFR inhibitor, fruquintinib can effectively block tumor angiogenesis and significantly improve patients’ PFS and OS. However, with the depth of the study, some new questions and research directions have emerged.

Firstly, although fruquintinib blocks tumor angiogenesis by inhibiting VEGFR-1, -2, and -3, its specific molecular mechanism has not been fully elucidated. An in-depth study of its mechanism of action, especially its behavior in different TMEs, could help develop more precise and effective therapeutic regimens. In addition, the development of combination therapies of fruquintinib with other targeted drugs or immunotherapeutic agents could further improve the therapeutic effect and overcome drug resistance.

Secondly, fruquintinib is now widely used as a third-line treatment for patients with mCRC after multiple treatment failures. Its efficacy and safety at this stage have laid the foundation for its use in second-line therapy. Future studies should focus on evaluating the efficacy of the fruquintinib combination strategy in second-line therapy, especially its advantages over existing standard second-line regimens such as the FOLFIRI regimen. In addition, there are clinical trials in second-line therapy aimed at investigating the efficacy of fruquintinib in the context of specific gene mutations (e.g., KRAS, NRAS, and BRAF mutations) and understanding the sensitivity of different subtypes to fruquintinib. In the future, as research progresses, biomarkers that predict efficacy and resistance can be identified through genomics and molecular biology, thus enabling individualized treatment regimens.

Thirdly, the safety of the long-term use of fruquintinib is particularly important in first-line therapy. In the current clinical application, AEs such as hypertension, proteinuria, and HFS are common. These AEs not only affect the quality of life of patients but may also lead to treatment discontinuation in some cases. Therefore, future research should focus on optimizing the dosage regimen and route of administration to develop new formulations with fewer AEs. For example, extended-release formulations or local delivery systems may significantly reduce systemic AEs and improve patient tolerance and compliance. Especially for these patients with poor health conditions, the conventional recommended dose may not be given, but reducing the dose of the drug needs to be decided after a comprehensive assessment of the patient’s physical condition at work by the clinician. In addition, while fruquintinib’s contribution to oncological treatment has been well studied, its optimal timing and its exact place in the standard chemotherapy sequence remain unclear and need to be explored and defined by further studies.

Lastly, although clinical trials provide efficacy and safety data, real-world data are more important for a comprehensive assessment of the clinical use of fruquintinib. In the future, real-world studies should be strengthened to assess the long-term effects on patient survival and quality of life through long-term follow-ups so that the use of the drug can be more standardized. Noteworthily, the importance of biomarker prediction should be emphasized. Monitoring changes in biomarkers can provide deeper insights into the drug’s mechanisms of action, efficacy, and prognosis. For example, fruquintinib may exhibit stronger anti-tumor effects in patients with high VEGFR expression; measuring changes in circulating tumor DNA (ctDNA) levels [46] and specific mutations (such as KRAS, BRAF) before and after treatment can assess fruquintinib’s impact on tumor burden and its effects on tumors with different genotypes. Immunohistochemistry or imaging analysis could be used to detect changes in immune cells (such as T cell macrophages) and vasculature in tumor tissues before and after treatment, thereby revealing fruquintinib’s regulatory effects on the TME.

In conclusion, fruquintinib, as a novel VEGFR inhibitor, has demonstrated great potential in CRC treatment. Through continuous studies into its mechanisms, clinical optimization, and implementation of individualized treatment strategies, fruquintinib is expected to further improve the survival rate and quality of life of CRC patients. In the future, through multidisciplinary collaboration and innovation, fruquintinib will surely bring new hope and treatment options to more cancer patients.

## Figures and Tables

**Table 1:** Comparison of anti-angiogenic drugs in CRC.

Drug	Target	Study Design	ORR	mPFS (Months)	mOS (Months)	Common AEs	≥Grade 3 AEs	Reference
Fruquintinib	VEGFR-1, 2 and 3	FRESCO study	4.7%	3.7	9.3	Hypertension (21.2%), Proteinuria (3.2%), HFS (10.8%)	61.2%	[28]
Bevacizumab	VEGF-A	AVF2107g trial	44.8%	10.6	20.3	Hypertension (22.4%), Leukopenia (37%), Diarrhea (32.4%)	84.9%	[29]
Regorafenib	VEGFR-1, 2 and 3 PDGFR, FGFR	CORRECT trial	1.0%	1.9	6.4	HFS (16.6%), Hypertension (7.2%), Fatigue (9.6%)	54.1%	[26]
Ramucirumab	VEGFR-2	RAINBOW trial	28%	4.4	9.6	Hypertension (14.1%), Neutropenia (40.7%), Fatigue (12%)	80%	[30]
Apatinib	VEGFR-2	Apatinib phase II study	2.8%	2.6	6.5	Hypertension (35.2%), Proteinuria (47.7%), HFS (27.8%)	54%	[31]

Notes: VEGFR, vascular endothelial growth factor receptor; VEGF, vascular endothelial growth factor; PDGFR, platelet-derived growth factor receptor; FGFR, fibroblast growth factor receptor; ORR, objective response rate; mPFS, median progression-free survival; mOS, median overall survival; AEs, adverse events; HFS, hand-foot syndrome.

**Table 2:** Published clinical trials of fruquintinib for mCRC.

Study Names	Study Location	No. of Patients	Inclusion Criteria	Median OS, Months (95%CI)	OS, HR (95%CI)	Median PFS, Months (95%CI)	PFS, HR (95%CI)	Reference
Phase I								
NCT01975077	China	42	Standard regimen failed or no standard regimen available; at least one measurable lesion (larger than 10 mm in diameter by spiral CT scan).	8.88 (7.53–15.53)	-	5.80 (4.01–7.60)	-	[38]
Phase II								
NCT02196688	China	71	Failed 2 or more lines of chemotherapy; at least one measurable lesion (larger than 10 mm in diameter by spiral CT scan).	7.72 (6.90–10.28) vs. 5.52 (3.61–11.30)	0.71 (0.38–1.34)	4.73 (2.86–5.59) vs. 0.99 (0.95–1.58)	0.30 (0.15–0.59)	[38]
NCT05025631	China	29	Patients who are refractory to or unfit for standard therapies; at least 4 weeks after the last anti-tumor therapy (chemotherapy, radiotherapy, biotherapy, or hormone therapy) and more than 3 months after operation treatment before enrollment. No other anti-tumor concomitant treatment (including steroid drugs).	7.6 (6.5–8.7)	-	3.8 (2.7–4.9)	-	[39]
Phase III								
FRESCO	China	416	Standard regimen failed or no standard regimen available.	9.30 (8.0–10.0) vs. 6.57 (5.97–7.62)	0.65 (0.51–0.83)	3.71 (2.79–4.63) vs. 1.84 (1.81–2.76)	0.26 (0.21–0.34)	[28]
FRESCO-2	Global	691	Treated with standard approved therapies: fluoropyrimidine-, oxaliplatin-, and irinotecan-based chemotherapy, an anti-VEGF biological therapy, and, if RAS wild-type, an anti-EGFR therapy.	7.4 (6.7–8.2) vs. 4.8 (4.0–5.8)	0.66 (0.55–0.80)	3.7 (3.5–3.8) vs. 1.8 (1.8–1.9)	0.32 (0.27–0.39)	[40]

Notes: OS, overall survival; PFS, progression-free survival; HR, hazard ratio; CI, confidence interval; CT, computed tomography; VEGF, vascular endothelial growth factor; RAS, renin-angiotensin system; EGFR, epidermal growth factor receptor.

**Table 3:** Ongoing clinical trials of fruquintinib for CRC.

NCT Number	Phase	Therapy Line	No. of Patients	Inclusion Criteria	Treatment Option	Primary Endpoint	Start Date	Study Type
NCT04296019	II	1	110	Patients with metastatic left-sided colon cancer with RAS mutation or right-sided colon cancer who achieved RECIST1.1-assessed SD (stable disease) or PR (partial response) or CR (complete response) after 18–24 weeks of first-line treatment.	Fruquintinib	PFS	1 February 2021	Randomized, controlled, multicenter
NCT05993702	-	3	45	Patients with mCRC who have failed two or more standard therapies.	TAS-102+regorafenib vs. TAS-102+fruquintinib	PFS	1 September 2023	Single-arm, multicenter, observational
NCT06115733	I/II	1	56	Patients with mCRC who achieved CR, PR, SD (RECIST 1.1) after standard first-line chemotherapy (FOLFOX, FOLFIRI, XELOX, FOLFOXIRI ± targeted therapy).	Capecitabine + fruquintinib	PFS, RP2D	28 December 2023	Single-arm, prospective
NCT06010888	II	1	92	Patients with mCRC who have not received systematic anti-tumor therapy before or who have received neoadjuvant/adjuvant therapy may be screened from the time of last chemotherapy to recurrence or progression of more than 6 months.	Fruquintinib + mFOLFOX6/FOLFIRI	ORR	31 October 2023	Single-arm, prospective
NCT05771181	II	-	25	Patients with mCRC who were progression or intolerant to prior standard therapy during or after standard therapy.	Tislelizumab, Fruquintinib, Vitamin E	ORR	1 March 2023	Single-arm, prospective
NCT06255379	II	3	52	Patients with mCRC who are refractory to at least second-line standard treatment containing fluorouracil, oxaliplatin, and irinotecan.	Fuquinitinib + Tegafur Gimeracil Oterac	PFS	6 May 2024	Single-arm, prospective, multicenter
NCT06356584	II	3	141	Patients with mCRC who previously received standard first- and second-line systemic anti-tumor therapy.	Sintilimab fruquintinib	PFS	1 April 2024	Randomized, prospective
NCT05842525	II	2	42	Patients with mCRC who had disease progression during or within 3 months of the last dose of first-line therapy.	Fruquintinib + FOLFIRI vs. Maintenance treatment	ORR	1 October 2023	Single-arm, prospective
NCT05661357	Ⅳ	3	51	Patients with mCRC who are pathologically diagnosed as HER-2 expression or mutation and who fail or are intolerant after second-line treatment.	Disitamab Vedotin + Fruquintinib	ORR	1 January 2023	Single-arm, prospective
NCT06497985	III	2	430	Patients with mCRC who are previously treated and have shown disease progression or could not tolerate standard treatment.	Tucidinostat + sintilimab + bevacizumab vs. fruquintinib	OS	30 September 2024	Randomized control, multicenter, prospective
NCT05634590	II	2	68	Patients with RAS-mutated mCRC who failed in previous standard therapy.	Fruquintinib + FOLFIRI/FOLFOX	PFS	1 December 2022	Single-arm, prospective
NCT05016869	I/ II	1	48	Patients with mCRC who have achieved CR, PR, or SD after up to 8 cycles of first-line standard treatment and remained unresectable.	Fruquintinib + capecitabine	RP2D, PFS	12 April 2022	Single-arm, prospective
NCT06347198	I	3	30	Patients with mCRC who are failure of prior second- and back-line standard therapy.	Fruquintinib + Sintilimab +inulin vs. Fruquintinib +Sintilimab	Intestinal microbiota	10 April 2024	Randomized control, prospective
NCT06423937	II	2	129	Patients with unresectable non-MSI-H mCRC with liver metastasis who had only received one standard first-line systemic treatment and were confirmed to be ineffective or could not tolerate first-line treatment.	Fruquintinib + Camrelizumab + HAIC vs. Fruquintinib + Camrelizumab	ORR	1 May 2024	Double-cohort, open, single-center
NCT06255379	II	3	52	Patients with mCRC who are refractory to at least second-line standard treatment containing fluorouracil, oxaliplatin, and irinotecan.	Fruquinitinib + Tegafur Gimeracil Oteracil	PFS	6 May 2024	Single-arm, open, multicenter
NCT06234007	II	3	45	Patients with locally advanced rectal adenocarcinoma who have not received any anti-tumor treatment.	Fruquintinib, Adebrelimab, Oxaliplatin, Capecitabine	pCR	1 December 2023	Single-arm, multicenter
NCT05451719	II	1	116	Patients with adenocarcinoma of the colon or rectum (stage IV) who have achieved disease control after 6 cycles of first-line standard chemotherapy and are still unresectable.	Ruquintinib + Capecitabine vs. Capecitabine	PFS	July 2022	Randomized, multicenter

Notes: PR, partial response; CR, complete response; SD, stable disease; mCRC, metastatic colorectal cancer; PFS, progression-free survival; OS, overall survival; RP2D, recommended phase 2 dose; ORR, objective response rate; pCR, pathological complete response; HER-2, human epidermal growth factor receptor 2; RAS, renin-angiotensin system; MSI-H, high microsatellite instability.

## Data Availability

All data are included in this manuscript.

## References

[1] Bray F., Laversanne M., Sung H., Ferlay J., Siegel R.L., Soerjomataram I., Jemal A. (2024). Global cancer statistics 2022: GLOBOCAN estimates of incidence and mortality worldwide for 36 cancers in 185 countries. CA A Cancer J. Clin..

[2] Han B., Zheng R., Zeng H., Wang S., Sun K., Chen R., Li L., Wei W., He J. (2024). Cancer incidence and mortality in China, 2022. J. Natl. Cancer Cent..

[3] Wang R., Lian J., Wang X., Pang X., Xu B., Tang S., Shao J., Lu H. (2023). Survival rate of colorectal cancer in China: A systematic review and meta-analysis. Front. Oncol..

[4] Pearson R.K. (2010). Cetuximab and Chemotherapy as Initial Treatment for Metastatic Colorectal Cancer. Yearb. Gastroenterol..

[5] Venook A.P., Niedzwiecki D., Lenz H.-J., Innocenti F., Fruth B., Meyerhardt J.A., Schrag D., Greene C., O’Neil B.H., Atkins J.N. (2017). Effect of First-Line Chemotherapy Combined With Cetuximab or Bevacizumab on Overall Survival in Patients With *KRAS* Wild-Type Advanced or Metastatic Colorectal Cancer: A Randomized Clinical Trial. JAMA.

[6] Wu L., Yan H., Qin Y., Huang M., Wang T., Jin Q., Wei W. (2024). Fruquintinib plus oxaliplatin combined with S-1 (SOX) as neoadjuvant therapy for locally advanced gastric cancer (GC) or gastro-oesophageal junction adenocarcinoma (GEJ): A multicentre, phase, II, single-arm, open-label clinical trial (FRUTINEOGA) protocol. BMJ Open.

[7] Zhang Y., Wang Z.X., Shen L., Li J., Huang J., Su W.G., Zhang D.S., Xu R.H. (2023). A phase Ib/II study of fruquintinib in combination with paclitaxel as the second-line therapy for advanced gastric cancer. Cancer Commun..

[8] Grothey A., Cutsem E.V., Sobrero A., Siena S., Falcone A., Ychou M., Humblet Y., Bouché O., Mineur L., Barone C. (2013). Regorafenib monotherapy for previously treated metastatic colorectal cancer (CORRECT): An international, multicentre, randomised, placebo-controlled, phase 3 trial. Lancet.

[9] Sun L., Huang S., Li D., Mao Y., Wang Y., Wu J. (2021). Efficacy and Safety of Fruquintinib Plus PD-1 Inhibitors Versus Regorafenib Plus PD-1 Inhibitors in Refractory Microsatellite Stable Metastatic Colorectal Cancer. Front. Oncol..

[10] Zhou S., Shao F., Xu Z., Wang L., Jin K., Xie L., Chen J., Liu Y., Zhang H., Ou N. (2017). A phase I study to investigate the metabolism, excretion, and pharmacokinetics of [14C]fruquintinib, a novel oral selective VEGFR inhibitor, in healthy Chinese male volunteers. Cancer Chemother. Pharmacol..

[11] Shirley M. (2018). Fruquintinib: First Global Approval. Drugs.

[12] Chen Z., Jiang L. (2019). The clinical application of fruquintinib on colorectal cancer. Expert Rev. Clin. Pharmacol..

[13] Cao J., Zhang J., Peng W., Chen Z., Fan S., Su W., Li K., Li J. (2016). A Phase I study of safety and pharmacokinetics of fruquintinib, a novel selective inhibitor of vascular endothelial growth factor receptor-1, -2, and -3 tyrosine kinases in Chinese patients with advanced solid tumors. Cancer Chemother. Pharmacol..

[14] Folkman J. (1971). Tumor Angiogenesis: Therapeutic Implications. N. Engl. J. Med..

[15] Shibuya M. (2011). Vascular Endothelial Growth Factor (VEGF) and Its Receptor (VEGFR) Signaling in Angiogenesis: A Crucial Target for Anti- and Pro-Angiogenic Therapies. Genes Cancer.

[16] Shaw P., Dwivedi S.K.D., Bhattacharya R., Mukherjee P., Rao G. (2024). VEGF signaling: Role in angiogenesis and beyond. Biochim. Biophys. Acta.

[17] Patell K., Mears V.L., Storandt M.H., Mahipal A. (2024). Metabolism, toxicity and management of fruquintinib: A novel drug for metastatic colorectal cancer. Expert Opin. Drug Metab. Toxicol..

[18] Olsson A.-K., Dimberg A., Kreuger J., Claesson-Welsh L. (2006). VEGF receptor signalling-in control of vascular function. Nat. Rev. Mol. Cell Biol..

[19] Simons M., Gordon E., Claesson-Welsh L. (2016). Mechanisms and regulation of endothelial VEGF receptor signalling. Nat. Rev. Mol. Cell Biol..

[20] Bui H.M., Enis D., Robciuc M.R., Nurmi H.J., Cohen J., Chen M., Yang Y., Dhillon V., Johnson K., Zhang H. (2016). Proteolytic activation defines distinct lymphangiogenic mechanisms for VEGFC and VEGFD. J. Clin. Investig..

[21] Wang L., Liu W.-Q., Broussy S., Han B., Fang H. (2024). Recent advances of anti-angiogenic inhibitors targeting VEGF/VEGFR axis. Front. Pharmacol..

[22] Choti M.A. (2004). Bevacizumab in combination with irinotecan plus fluorouracil plus leucovorin chemotherapy prolongs survival but increases adverse events in people with metastatic colorectal cancer. Cancer Treat. Rev..

[23] Lu S., Zhou J.-Y., Niu X.-M., Zhou J.-Y., Jian H., Yin H.-Y., Guan S., Wang L.-F., Li K., He J. (2021). Fruquintinib with gefitinib as first-line therapy in patients carrying EGFR mutations with advanced non-small cell lung cancer: A single-arm, phase II study. Transl. Lung Cancer Res..

[24] Grandinetti C.A., Goldspiel B.R. (2007). Sorafenib and Sunitinib: Novel Targeted Therapies for Renal Cell Cancer. Pharmacother. J. Hum. Pharmacol. Drug Ther..

[25] Keating G.M., Santoro A. (2009). Sorafenib: A Review of its Use in Advanced Hepatocellular Carcinoma. Drugs.

[26] Nuray Sever Ö., Aktaş G., Aksoy B., Yıldırım M. (2022). Clinical factors predicting response to regorafenib in metastatic colorectal cancer. Euroasia J..

[27] Van Geel R.M.J.M., Beijnen J.H., Schellens J.H.M. (2012). Concise Drug Review: Pazopanib and Axitinib. Oncol..

[28] Li J., Qin S., Xu R.-H., Shen L., Xu J., Bai Y., Yang L., Deng Y., Chen Z.-D., Zhong H. (2018). Effect of Fruquintinib vs Placebo on Overall Survival in Patients With Previously Treated Metastatic Colorectal Cancer: The FRESCO Randomized Clinical Trial. JAMA.

[29] Hurwitz H., Fehrenbacher L., Novotny W., Cartwright T., Hainsworth J., Heim W., Berlin J., Baron A., Griffing S., Holmgren E. (2004). Bevacizumab plus irinotecan, fluorouracil, and leucovorin for metastatic colorectal cancer. N. Engl. J. Med..

[30] Wilke H., Muro K., Van Cutsem E., Oh S.-C., Bodoky G., Shimada Y., Hironaka S., Sugimoto N., Lipatov O., Kim T.-Y. (2014). Ramucirumab plus paclitaxel versus placebo plus paclitaxel in patients with previously treated advanced gastric or gastro-oesophageal junction adenocarcinoma (RAINBOW): A double-blind, randomised phase 3 trial. Lancet Oncol..

[31] Li J., Qin S., Xu J., Xiong J., Wu C., Bai Y., Liu W., Tong J., Liu Y., Xu R. (2016). Randomized, Double-Blind, Placebo-Controlled Phase III Trial of Apatinib in Patients With Chemotherapy-Refractory Advanced or Metastatic Adenocarcinoma of the Stomach or Gastroesophageal Junction. J. Clin. Oncol..

[32] Zhang Y., Zou J.-Y., Wang Z., Wang Y. (2019). Fruquintinib: A novel antivascular endothelial growth factor receptor tyrosine kinase inhibitor for the treatment of metastatic colorectal cancer. Cancer Manag. Res..

[33] Sun Q., Zhou J., Zhang Z., Guo M., Liang J., Zhou F., Long J., Zhang W., Yin F., Cai H. (2014). Discovery of fruquintinib, a potent and highly selective small molecule inhibitor of VEGFR 1, 2, 3 tyrosine kinases for cancer therapy. Cancer Biol. Ther..

[34] Li Q., Cheng X., Zhou C., Tang Y., Li F., Zhang B., Huang T., Wang J., Tu S. (2022). Fruquintinib Enhances the Antitumor Immune Responses of Anti-Programmed Death Receptor-1 in Colorectal Cancer. Front. Oncol..

[35] Liu X., Guo A., Tu Y., Li W., Li L., Liu W., Ju Y., Zhou Y., Sang A., Zhu M. (2020). Fruquintinib inhibits VEGF/VEGFR2 axis of choroidal endothelial cells and M1-type macrophages to protect against mouse laser-induced choroidal neovascularization. Cell Death Dis..

[36] Lavacchi D., Roviello G., Guidolin A., Romano S., Venturini J., Caliman E., Vannini A., Giommoni E., Pellegrini E., Brugia M. (2023). Evaluation of Fruquintinib in the Continuum of Care of Patients with Colorectal Cancer. Int. J. Mol. Sci..

[37] Gu Y., Wang J., Li K., Zhang L., Ren H., Guo L., Sai Y., Zhang W., Su W. (2014). Preclinical pharmacokinetics and disposition of a novel selective VEGFR inhibitor Fruquintinib (HMPL-013) and the prediction of its human pharmacokinetics. Cancer Chemother. Pharmacol..

[38] Xu R.-H., Li J., Bai Y., Xu J., Liu T., Shen L., Wang L., Pan H., Cao J., Zhang D. (2017). Safety and efficacy of fruquintinib in patients with previously treated metastatic colorectal cancer: A phase Ib study and a randomized double-blind phase II study. J. Hematol. Oncol..

[39] Tan S., Zhang S., Zhou N., Cai X., Yi C., Gou H. (2023). Efficacy and safety of fruquintinib dose-escalation strategy for elderly patients with refractory metastatic colorectal cancer: A single-arm, multicenter, phase II study. Cancer Med..

[40] Dasari A., Lonardi S., Garcia-Carbonero R., Elez E., Yoshino T., Sobrero A., Yao J., García-Alfonso P., Kocsis J., Cubillo Gracian A. (2023). Fruquintinib versus placebo in patients with refractory metastatic colorectal cancer (FRESCO-2): An international, multicentre, randomised, double-blind, phase 3 study. Lancet.

[41] Liu S., Lu L., Pan F., Yang C., Liang J., Liu J., Wang J., Shen R., Xin F.-Z., Zhang N. (2022). Real-World Data: Fruquintinib in Treating Metastatic Colorectal Cancer. Oncol. Res..

[42] Deng Y.-Y., Zhang X.-Y., Zhu P.-F., Lu H.-R., Liu Q., Pan S.-Y., Chen Z.-L., Yang L. (2023). Comparison of the efficacy and safety of fruquintinib and regorafenib in the treatment of metastatic colorectal cancer: A real-world study. Front. Oncol..

[43] Wang F., Shen L., Guo W., Liu T., Li J., Qin S., Bai Y., Chen Z., Wang J., Pan Y. (2024). Fruquintinib plus paclitaxel versus placebo plus paclitaxel for gastric or gastroesophageal junction adenocarcinoma: The randomized phase 3 FRUTIGA trial. Nat. Med..

[44] Li J., Xu R., Bai Y., Xu J., Liu T., Shen L., Wang L., Pan H., Fan S., Hua Y. (2015). A randomized, double-blind, placebo-controlled, multicenter Phase II clinical trial of fruquintinib in patients with metastatic colorectal cancer (mCRC). Eur. J. Cancer.

[45] Peterson D.E., Boers-Doets C.B., Bensadoun R.J., Herrstedt J. (2015). Management of oral and gastrointestinal mucosal injury: ESMO Clinical Practice Guidelines for diagnosis, treatment, and follow-up. Ann. Oncol..

[46] Wan J.C.M., Massie C., Garcia-Corbacho J., Mouliere F., Brenton J.D., Caldas C., Pacey S., Baird R., Rosenfeld N. (2017). Liquid biopsies come of age: Towards implementation of circulating tumour DNA. Nat. Rev. Cancer.

